# Association between Vitamin B and Obesity in Middle-Aged and Older Chinese Adults

**DOI:** 10.3390/nu15030483

**Published:** 2023-01-17

**Authors:** Yu Fu, Zhanyong Zhu, Zhaolan Huang, Ruikun He, Ying Zhang, Yuanyuan Li, Wei Tan, Shuang Rong

**Affiliations:** 1 Academy of Nutrition and Health, Hubei Province Key Laboratory of Occupational Hazard Identification and Control, School of Public Health, Wuhan University of Science and Technology, Wuhan 430065, China; 2 Department of Plastic Surgery, Renmin Hospital of Wuhan University, Wuhan 430060, China; 3 Geriatric Hospital Affiliated to Wuhan University of Science and Technology, Wuhan 430065, China; 4 BYHEALTH Institute of Nutrition & Health, Huangpu District, Guangzhou 510663, China; 5 Department of Nutrition, School of Public Health, Wuhan University, Wuhan 430071, China

**Keywords:** obesity, vitamin B (B_1_, B_2_, B_6_, and B_9_), middle-aged and older adults

## Abstract

Objective: Previous studies have found that obese people have lower levels of vitamin B, but most have focused on obesity as defined by body mass index (BMI), and its relationship with other types of obesity is unclear. The aim of this study was to explore the relationship between vitamin B levels and obesity assessed by different definitions among Chinese middle-aged and older community-dwelling adults. Methods: This cross-sectional study included 887 participants aged 45 years and older (45–82 years). The concentrations of vitamin B (B_1_, B_2_, B_6_, and B_9_) were measured by robotic dry blood spot extraction systems in combination with liquid chromatography–tandem mass spectrometry. BMI, body fat percentage (BF%), visceral fat area (VFA), and waist circumference (WC) were used to diagnose obesity. VFA and BF% were assessed by bioelectrical impedance analysis. The logistic regression model was used to assess the associations between vitamin B levels and the odds of obesity. Results: The average age of all participants was 60.77 (SD 6.33) years. The prevalence of obesity varied from 8.6% to 52.4% depending on different diagnostic criteria. After adjusting for covariates, a negative correlation was observed between vitamin B_1_ level and obesity according to the criteria of WC, VFA, and BF%, and the adjusted odds ratio (OR) was 0.47, 0.52, and 0.46, respectively. When using WC and BF% to define obesity, higher quartiles of vitamin B_2_ were negatively associated with the odds of obesity (OR: 0.62 and 0.62, respectively). Vitamin B_6_ was inversely associated with VFA-defined and BF%-defined obesity (OR: 0.64 and 0.64, respectively). When using VFA and BF% to define obesity, a negative correlation was observed in vitamin B_9_ (OR: 0.61 and 0.67, respectively). Conclusions: Vitamin B (B_1_, B_2_, B_6_, and B_9_) level was negatively related to obesity (defined by WC, VFA, or BF%) in Chinese middle-aged and older adults.

## 1. Introduction

Obesity, as a global pandemic, is associated with increased adverse health outcomes such as mortality, cancer, cardiovascular diseases, dementia, and other chronic diseases [1]. With its fast rising prevalence, obesity brings a heavy burden on the healthcare system [2]. Obesity is a long-term nutritional disorder; in addition to the presence of excess energy, obese individuals also have vitamin deficiencies, especially of fat-soluble vitamins, folic acid, vitamin B_12_, and vitamin C [3].

Vitamin B may play an important role in the development of obesity and other metabolic diseases by affecting energy metabolism, oxidative stress, inflammatory response, and lipid metabolism [4,5,6,7,8]. Although studies have found a seemingly linked relationship between vitamin B and obesity, studies on the blood concentration of vitamin B and obesity are limited and inconsistent, and previous studies have mostly focused on vitamin B_9_ and vitamin B_12._ A case-control study reported that low serum folate (vitamin B_9_) levels are related to higher body mass index (BMI) and abdominal fat [9]. A systematic review of folate found there is no statistically significant association between folic acid (vitamin B_9_) and BMI [10]. In addition, a cross-sectional study of the Emirati population revealed that plasma concentrations of pyridoxal (vitamin B_6_) were much greater in obese people than in healthy ones [11]. Therefore, the relationship between vitamin B and obesity needs more investigation.

Obesity is defined as a disease process characterized by excessive body fat accumulation with multiple organ-specific consequences [12]. BMI is the most widely used tool for obesity diagnosis in epidemiology studies [13]. However, BMI only reflects overall obesity, not body fat distribution. Mounting evidence shows that body fat distribution is also associated with an increased risk of disease [14,15,16] and mortality [17]. Fat distribution has typically been expressed as waist circumference (WC) or visceral fat area (VFA). Body fat percentage (BF%) is also used to diagnose obesity, reflecting body fat mass. Previous studies have revealed the associations of vitamin B with BMI-defined obesity [18,19,20]; however, such questions have received little attention according to different obesity standards.

This study aimed to investigate the associations between vitamin B (B_1_, B_2_, B_6_ and B_9_) concentrations and obesity (defined by BMI, WC, VFA, and BF%, respectively) in middle-aged and older Chinese adults. The findings could enrich the research on vitamin B in various types of obesity, and could contribute to the prevention and management of obesity.

## 2. Methods

### 2.1. Participants

**Figure 1:**
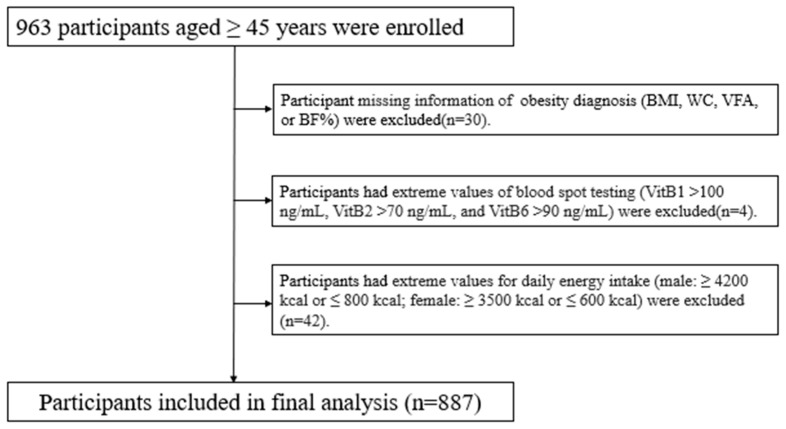
Flowchart of the inclusion of the study participants.

Participants in this study were recruited through the baseline of the Lifestyle and Healthy Aging of Chinese Square Dancer Study. Inclusion criteria were as follows: (i) Chinese; (ii) age ≥45 years; (iii) offered blood samples and completed dry blood spot testing; (iv) no participation in any clinical trial in the three months prior to the survey. Exclusion criteria were as follows: (i) were deaf or mute; (ii) had serious mental disease; (iii) were in the acute phase of severe diseases (e.g., cardiovascular and cerebrovascular diseases, cancer, and/or psychiatric diseases). Finally, 963 participants aged 45 and older were recruited. The study was approved by the Ethics Committee of Wuhan University of Science and Technology (201925), and obtained informed consent from all participants. A flow chart of the participants included in the current analysis is shown in Figure 1. Participants were excluded if they lacked information about obesity diagnosis (*n* = 30), had extreme values of blood spot testing (*n* = 4), or had extreme daily energy intake (*n* = 42). The final analysis comprised 887 participants (Figure 1).

### 2.2. Dry Blood Spot Collection and Laboratory Testing

The vitamin B concentrations in whole blood were gathered by the dry blood spot method (DBS). The blood samples were dropped on a special filter paper card, dried, and stored separately in a Ziploc bag with a desiccant. DBS samples were stored at −20 °C for one month before being returned to the Institute of Nutrition and Health, Wuhan University of Science and Technology. DBS samples were detected for discoloration or the absence of desiccant before analysis. Multivitamins in DBS were detected using the method of Lin Y et al. [21]. This method can detect multivitamins in the blood in a short time and solve the technical defects in the current detection methods of multivitamins. Dry blood spot samples were treated with a DBS-MS 500 (CAMAG, Muttenz, Switzerland). It can automatically pick up DBS cards, extract them after the identification check, and then measure them by liquid chromatography–tandem mass spectrometry. All samples were analyzed using a SCIEX ExionLC system coupled with a SCIEX Triple Quad 6500 mass spectrometer. Individuals with extreme values of vitamin B concentrations (VitB_1_ > 100 ng/mL, VitB_2_ > 70 ng/mL, and VitB_6_ > 90 ng/mL) were excluded.

### 2.3. Body Composition and Obesity Ascertainment

Each participant underwent a standardized physical examination with measurements of height, waist circumference (WC), and blood pressure. Height and WC were obtained by a standardized physical examination, and participants wore light clothing and no shoes. Bioelectrical impedance analysis (BIA) (H-key350 version, Seehigher Company, Beijing, China) was used to assess body composition, including weight, body fat percentage (BF%), and visceral fat area (VFA). BMI was calculated as weight in kilograms divided by the square of height in meters (kg/m^2^). We used different indicators to define obesity, including BMI, WC, VFA, and BF%. BMI ≥ 28 kg/m^2^ was considered as general obesity [22]. Women with a WC greater than or equal 85 cm and men with a WC greater than or equal 90 cm were classified as having central obesity [23]. VFA was used to characterize visceral adiposity (VFA > 100 cm^2^) [24]. BF% was used to describe obesity with excess body fat (BF% ≥ 35% in women and ≥25% in men) [25]. Information on body composition and obesity determination is presented in the Table 1.

### 2.4. Covariates

Sociodemographic information and behavioral characteristics were obtained by standardized questionnaires. Sociodemographic information included age (continuous variable), sex (female or male), marital status (married, single, separated, or divorced), education (less than middle school, middle and high school, or college or higher), annual household income per capita (<20,000, 20,000–40,000, or >40,000), and employment status (retired or paid employment). Behavioral characteristics included smoking status (current smoker or non-smoker), alcohol status (current drinker or non-drinker), and physical activity. The Physical Activity Scale for the Elderly (PASE) was used to determine if the physical activity in the past week met the recommendations of the 2020 WHO Guidelines on Physical Activity and Sedentary Behavior [26]. The dietary intake was evaluated by using a 66-item semi-quantitative food frequency questionnaire. The participants were asked the frequency and average intake of each food during the last year. According to the collected data, daily total energy intake was calculated with the use of the China Food Composition (book 1, 2nd edition). Participants who had extreme daily energy intake were excluded (i.e., energy intake more than 4200 kcal or less than 800 kcal for males, and energy intake more than 3500 kcal or less than 600 kcal for females). We also examined participants' use of nutritional supplements (yes/no). Disease information included cardiovascular disease (self-reported or physician diagnoses), cancer (self-reported or physician diagnoses), hyperlipidemia (self-report or physician diagnoses), hypertension (self-reported and/or systolic blood pressure ≥140 mmHg and/or diastolic blood pressure ≥90 mmHg), and diabetes (self-reported and/or fasting blood glucose ≥7.0 mmol/L and/or random blood glucose ≥11.1 mmol/L). These diseases are considered possible confounders, and having more than one is defined as having a chronic disease.

### 2.5. Statistical Analysis

The concentrations of vitamin B (B_1_, B_2_, B_6_ and B_9_) were divided into quartiles. Continuous variables were presented as the mean (standard deviation (SD)), categorical variables were presented as the numbers (percentages). Vitamin B concentration was measured by median and interquartile spacing. We used a nonparametric test (Wilcoxon test) to compare median vitamin B concentration between participants with and without obesity. Binary logistic regression was performed to calculate the odds ratio (OR) and 95% confidence intervals (CIs) between vitamin B concentrations and obesity under different definitions. The association between vitamin B concentrations and obesity was examined using binary logistic regression. The odds ratios (ORs) and 95% confidence intervals (CIs) were computed for each quartile of vitamin B by considering the lowest quartile as the reference group. We first calculated the crude association without adjusting for any covariates. Then, age, sex, marital status, education, smoking status, drinking status, physical activity, family income, daily energy intake (tertiles), and chronic diseases were included in the final adjusted models. Secondary analysis was performed to test the robustness of our results. We performed subgroup analyses across sex and age (≤60 vs. >60 years). All the analyses were conducted using SAS version 9.4 (SAS Institute Inc., Cary, NC, USA), and a two-sided *p*-value < 0.05 was considered statistically significant.

## 3. Results

### 3.1. Participants’ Characteristics

This study included 887 participants (93.7% were female). The ages ranged from 45 to 82 years, with a mean age of 60.8 years (SD, 6.33 years). The main characteristics of the population are presented in Table 2: 96.3% of participants were never or former smokers, 83.4% were never or former drinkers, 82.0% met the recommended level of physical activity, and 81.3% were retired. According to different obesity diagnostic methods, the obesity prevalence was 8.6% (BMI), 33.7% (WC), 52.4% (VFA), and 48.6% (BF%), respectively.

### 3.2. Vitamin B Concentrations under Different Definitions of Obesity

Table 3 shows the median concentrations of vitamin B_1_, vitamin B_2_, vitamin B_6_ and vitamin B_9_ detected by the DBS method in the obese and non-obese groups under different diagnostic criteria. Except for BMI, there was a significant difference in vitamin B_1_ concentrations between the obese and non-obese groups when using different obesity indicators (VFA, WC, and BF%). The median concentrations of vitamin B_2_ differed significantly between the obese and non-obese groups when using BMI, WC, or BF% to diagnose obesity.

### 3.3. The Relationships between Blood Vitamin B and Four Measurements of Obesity

Vitamin B concentrations measured by the DBS method were associated with obesity when using VFA, WC, and BF% to diagnose obesity. As shown in Table 4, in the fully adjusted models, compared with the lowest quartile of vitamin B_1_ concentrations (Q1), the OR of central obesity for the Q4 was 0.47 (95% CI: 0.30, 0.73). When vitamin B_1_ concentrations were at Q3 and Q4, the adjusted ORs of VFA-defined obesity were 0.58 (95% CI: 0.39, 0.86) and 0.52 (95% CI: 0.35, 0.77), respectively (*p* for trend < 0.05). When vitamin B_1_ concentrations were at Q2, Q3, and Q4, the adjusted ORs of BF%-defined obesity were 0.66 (95% CI: 0.44, 0.98), 0.55 (95% CI: 0.37, 0.82), and 0.46 (95% CI: 0.30, 0.69), respectively (*p* for trend < 0.05).

Vitamin B_2_ concentrations at Q4 was also a protective factor for central obesity (OR = 0.62, 95% CI: 0.40, 0.94). Compared with the lowest quartile of vitamin B_2_ concentrations, the adjusted odds of BF%-based obesity for the Q2 and Q4 were 0.65 (0.44, 0.97) and 0.62 (0.41, 0.92), respectively. The concrete results are presented in Table 5.

When vitamin B_6_ concentrations were at Q4, in the fully adjusted models, the ORs (95% CI) of VFA-based obesity and BF%-based obesity were 0.64 (0.43, 0.95) and 0.64 (0.43, 0.96), respectively (Table 6).

As is demonstrated in Table 7, when vitamin B_9_ concentrations were at Q2 and Q4, the adjusted odds of VFA-defined obesity were 0.67 (95% CI: 0.45, 0.99) and 0.61 (95% CI: 0.41, 0.91), respectively. Vitamin B_9_ concentrations at Q4 was also a protective factor for BF%-defined (OR = 0.67, 95% CI: 0.45, 0.99).

### 3.4. Secondary Analysis

Among the older adults (≥60 years), the highest quartile of vitamin B_1_, vitamin B_6_, and vitamin B_9_ was negatively correlated with visceral obesity (Supplementary Table S1). A negative association was observed between vitamin B_1_ and vitamin B_2_ and central obesity (Supplementary Table S2). Vitamin B_1_, vitamin B_2_, and vitamin B_6_ were associated with BF%-defined obesity (Supplementary Table S3). Moreover, we observed an association between vitamin B and obesity in females, but not in males. Vitamin B_1_, vitamin B_6_, and vitamin B_9_ concentrations were significantly associated with odds of visceral obesity (Supplementary Table S4). Vitamin B_1_ and vitamin B_2_ were associated with BF%-defined obesity (Supplementary Table S5). An inverse association between Vitamin B_1_ and central obesity was observed (Supplementary Table S6).

### 3.5. Discussion

In this cross-sectional study, we examined the relationship between vitamin B concentrations and obesity, defined according to BMI, WC, VFA, and BF% in middle-aged and older Chinese adults. Our findings showed that vitamin B_1_ concentrations were negatively correlated with obesity (WC, VFA, and BF%), and vitamin B_2_ concentrations were negatively related with WC- and BF%-defined obesity. When using VFA or BF% to define obesity, both vitamin B_6_ and vitamin B_9_ were negatively associated with obesity. There was no statistically significant association between vitamin B and BMI-based obesity.

Obese people regularly consume high-calorie, low-nutrient food, which can easily cause insufficient nutritional intake. Their obesity status will also affect the absorption and metabolism of vitamins. Vitamin B is involved in many important physiological processes, and deficiencies can cause different symptoms [27]. Research on blood vitamin B and obesity is limited and inconsistent. Previous studies also found that vitamin B is associated with BMI, while ours did not. A case-control study showed that serum folate levels were lower in obese subjects (BMI > 25 kg/m^2^) than in healthy controls, and serum folate was associated with BMI and waist to hip ratio [9]. In a study of postmenopausal women, obese women (BMI > 30 kg/m^2^) had lower serum folate concentrations than normal-weight women [28], and serum folate was related to BMI and BF%. A cross-sectional study of Mexican-American children indicated that serum folate concentrations were negatively correlated with BMI and fat mass [20]. Another study used five measurements to define obesity (BMI, WC, waist-to-height ratio, a body shape index, and body roundness index) and found that low levels of serum folic were associated with an increased odds of five types of obesity among middle-aged Koreans [29]. Alternatively, a cross-sectional study with 57 healthy and 57 obese Emirati volunteers revealed that plasma pyridoxal levels were higher in obese participants than in healthy ones [11]. The discrepancy in the results may be attributable to differences in the participants’ characteristics, measurement of vitamin B, sample size, and the cut-off point of BMI. Our study adds to the evidence for a relationship between different B vitamins and different types of obesity.

The mechanisms between vitamin B levels and obesity have not been elucidated. Several possible pathways have been proposed to explain the observed association. First, vitamin B plays an important role in energy metabolism [4,30], and its deficiency may impair energy metabolism and stimulate fat production, and lead to obesity. A study found that vitamin B also appears to improve metabolism [31]. Second, vitamin B has antioxidant and anti-inflammatory effects [32,33,34,35]. Vitamin deficiencies can lead to increased oxidative stress and inflammation. Moreover, a study found that intestinal oxidative stress may lead to reduced intestinal absorption of dietary folic acid [36], resulting in folic acid deficiency and forming a vicious cycle. Third, deficiency of folic acid and vitamin B_6_ may raise homocysteine (HCY) concentrations, and HCY inhibits lipolysis by activating the AMP-activated protein kinase pathway [37]. Fourth, obesity is associated with a chronic, low-grade inflammatory state; inflammation could affect vitamin B_6_ metabolism in different tissues [38]. An animal study found that orotic acid induced fatty liver affected vitamin B concentrations in rats, resulting in increased urine excretion of vitamin B_1_, pantothenic acid, folate, and biotin. Further studies are needed to verify this association between different definitions of obesity and vitamin B level.

### 3.6. Strength and Limitation

This study has some strengths. This is the first study to evaluate the association of vitamin B concentrations and different definitions of obesity. Second, major results were still observed in women and elderly participants, especially the association of vitamin B_1_ with obesity (WC, VFA, or BF%). There are also several limitations in this study. First, our study used the DBS method to detect the concentration of vitamin B in whole blood instead of serum, so the relationship between serum vitamin B and obesity needs to be further studied. Second, given that the study was cross-sectional and cannot infer cause-and-effect, there may also be an inverse relationship between serum B vitamins and obesity. Third, we did not examine the participants’ levels of inflammation or thyroid function, which might also affect vitamin B levels in the body. Fourth, the main participants were female, so we should be cautious when extrapolating the results to other populations.

## 4. Conclusions

The study demonstrated that vitamin B (B_1_, B_2_, B_6_ and B_9_) was negatively related to obesity when using WC, VFA, and BF% to define obesity. Higher vitamin B levels may improve body fat distribution and reduce fat mass among adults aged 45 years and older. These results suggest that, compared with general obesity, more attention should be paid to vitamin B levels in people with different obesity characteristics in order to prevent and manage obesity.

## Figures and Tables

**Table 1:** Measurement of body composition and diagnosis of obesity.

Method of Examination of Body Composition	
Method	Body composition
Physical examination	height, waist circumference, blood pressure
Bioelectrical impedance analysis	weight, body fat percentage, visceral fat area
Diagnosis of obesity	
Obesity indicators	Diagnosis
BMI	BMI ≥ 28 kg/m^2^
Waist circumference (WC)	male: ≥ 90 cm; female: ≥ 85 cm
Visceral fat area (VFA)	VFA > 100 cm
Body fat percentage (BF%)	male: ≥ 25%; female: ≥ 35%

**Table 2:** Distribution of all participants according to general characteristics.

Variables	
Age, years, mean (SD)	60.77 (6.33)
Female (%)	831 (93.69)
Marital status, n (%)	
Married	781 (88.05)
Other ^a^	106 (11.95)
Educational level, n (%)	
Primary school and below	77 (8.68)
Middle school and high school	697 (78.58)
College or above	113 (12.74)
Annual household income per capita, n (%)	
<20,000 CNY	229 (25.82)
20,000–40,000 CNY	421 (47.46)
>40,000 CNY	237 (26.72)
Smoking, n (%)	
Never or former smoker	854 (96.28)
Current smoker	33 (3.72)
Alcohol intake, n (%)	
Never or former drinker	740 (83.43)
Current drinker	147 (16.57)
Meeting physical activity recommendation, n (%)	727 (81.96)
Chronic diseases, n (%)	495 (55.81)
Retired, n (%)	721 (81.29)
General obesity ^b^, n (%)	76 (8.57)
Central obesity ^c^, n (%)	299 (33.71)
Obesity (VFA) ^d^, n (%)	465 (52.42)
Obesity (BF%) ^e^, n (%)	431 (48.59)

Data were expressed as the mean (SD) or n (%). Abbreviations: SD, standard deviation; VFA, visceral fat area; BF%, body fat percentage. ^a^ Other marital status included single, divorced, or widowed. ^b^ Obesity was defined as body mass index ≥ 28 kg/m^2^. ^c^ Obesity was defined as waist circumference (male > 90 cm, female > 85 cm). ^d^ Obesity was defined as VFA > 100 cm^2^. ^e^ Obesity was defined as BF% (male ≥ 25, female ≥ 35).

**Table 3:** Median Vitamin B concentration under different definitions of obesity (ng/mL) (M (P25, P75)).

	General Obesity ^a^		Central Obesity ^b^		Obesity (VFA) ^c^		Obesity (BF%) ^d^	
	No	Yes	No	Yes	No	Yes	No	Yes
VB_1_ (ng/mL)	3.2 (2.6, 4.4)	3.0 (2.5, 4.23)	3.4 (2.6, 4.5)	2.9 (2.5, 4.2) *	3.6 (2.7, 4.5)	3 (2.5, 4.3) *	3.6 (2.7, 4.5)	2.9 (2.5, 4.2) *
VB_2_ (ng/mL)	4.7 (3.0, 7.2)	3.8 (2.7, 5.7) *	4.7 (3.1, 7.4)	4.1 (2.8, 6.5) *	4.7 (3.1, 7.6)	4.5 (2.9, 6.9)	4.7 (3.1, 7.8)	4.3 (2.9, 6.8) *
VB_6_ (ng/mL)	12.3 (8.9, 17.4)	13.0 (10.1, 18.6)	12.3 (9.0, 17.9)	12.7 (9.0, 17.3)	12.5 (9.6, 18.6)	12.2 (8.7, 17.0)	12.3 (9.0, 17.9)	12.7 (9.0, 17.3)
VB_9_ (ng/mL)	4.3 (2.6, 6.6)	4.3 (2.9, 7.2)	4.3 (2.7, 6.6)	4.2 (2.5, 6.6)	4.4 (2.8, 6.8)	4.1 (2.4, 6.2) *	4.4 (2.8, 6.7)	4.1 (2.4, 6.3) *

Abbreviations: VFA, visceral fat area; BF%, body fat percentage. ^a^ Obesity was defined as BMI ≥ 28 kg/m^2^. ^b^ Obesity was defined as waist circumference (male > 90 cm, female > 85 cm). ^c^ Obesity was defined as VFA > 100 cm^2^. ^d^ Obesity was defined as BF% (male ≥ 25, female ≥ 35). * *p* < 0.05 compared with non-obese group.

**Table 4:** Association between vitamin B_1_ and obesity according to different obesity definitions.

	VB_1_				
	Quartile 1 (<2.60 ng/mL)	Quartile 2 (~3.16 ng/mL)	Quartile 3 (~4.38 ng/mL)	Quartile 4 (>4.38 ng/mL)	*p* for Trend
General obesity (BMI)					
Model 1 ^a^	1 (ref)	0.99 (0.53, 1.88)	0.84 (0.44, 1.62)	0.74 (0.38, 1.46)	0.328
Model 2 ^b^	1 (ref)	0.99 (0.52, 1.90)	0.82 (0.42, 1.61)	0.71 (0.35, 1.45)	0.298
Central obesity (WC)					
Model 1 ^a^	1 (ref)	0.89 (0.61, 1.30)	0.70 (0.47, 1.03)	0.46 (0.30, 0.69)	<0.001
Model 2 ^b^	1 (ref)	0.89 (0.59, 1.33)	0.71 (0.47, 1.07)	0.47 (0.30, 0.73)	<0.001
Obesity (VFA)					
Model 1 ^a^	1 (ref)	0.75 (0.51, 1.09)	0.57 (0.39, 0.83)	0.53 (0.36, 0.78)	<0.001
Model 2 ^b^	1 (ref)	0.68 (0.46, 1.01)	0.58 (0.39, 0.86)	0.52 (0.35, 0.77)	0.001
Obesity (BF%)					
Model 1 ^a^	1 (ref)	0.67 (0.46, 0.98)	0.53 (0.37, 0.78)	0.43 (0.29, 0.62)	<0.001
Model 2 ^b^	1 (ref)	0.66 (0.44, 0.98)	0.55 (0.37, 0.82)	0.46 (0.30, 0.69)	<0.001

Values are odds ratio (95% confidence interval). Abbreviations: BMI, body mass index; WC, waist circumference; VFA, visceral fat area; BF%, body fat percentage. ^a^ Model 1: unadjusted. ^b^ Model 2: adjusted for age, sex, marital status, education, smoking status, drinking status, employment status, physical activity, family income, daily energy intake (tertiles), nutritional supplement, and chronic diseases.

**Table 5:** Association between vitamin B_2_ and obesity according to different obesity definitions.

	VB_2_				
	Quartile 1 (<2.98 ng/mL)	Quartile 2 (~4.58 ng/mL)	Quartile 3 (~7.18 ng/mL)	Quartile 4 (>7.18 ng/mL)	*p* for Trend
General obesity (BMI)					
Model 1 ^a^	1 (ref)	0.95 (0.52, 1.74)	0.55 (0.28, 1.10)	0.60 (0.30, 1.17)	0.053
Model 2 ^b^	1 (ref)	0.97 (0.52, 1.80)	0.53 (0.26, 1.08)	0.53 (0.26, 1.06)	0.027
Central obesity (WC)					
Model 1 ^a^	1 (ref)	0.82 (0.56, 1.20)	0.70 (0.47, 1.03)	0.65 (0.44, 0.97)	0.022
Model 2 ^b^	1 (ref)	0.88 (0.58, 1.32)	0.75 (0.50, 1.14)	0.62 (0.40, 0.94)	0.019
Obesity (VFA)					
Model 1 ^a^	1 (ref)	0.94 (0.65, 1.36)	0.94 (0.65, 1.36)	0.78 (0.54, 1.14)	0.223
Model 2 ^b^	1 (ref)	0.94 (0.64, 1.39)	0.99 (0.67, 1.46)	0.74 (0.50, 1.10)	0.180
Obesity (BF%)					
Model 1 ^a^	1 (ref)	0.64 (0.44, 0.93)	0.80 (0.55, 1.16)	0.63 (0.43, 0.92)	0.053
Model 2 ^b^	1 (ref)	0.65 (0.44, 0.97)	0.88 (0.60, 1.31)	0.62 (0.41, 0.92)	0.078

Values are odds ratio (95% confidence interval). Abbreviations: BMI, body mass index; WC, waist circumference; VFA, visceral fat area; BF%, body fat percentage. ^a^ Model 1: unadjusted. ^b^ Model 2: adjusted for age, sex, marital status, education, smoking status, drinking status, employment status, physical activity, family income, daily energy intake (tertiles), nutritional supplement, and chronic diseases.

**Table 6:** Association between vitamin B_6_ and obesity according to different obesity definitions.

	VB_6_				
	Quartile 1 (<8.99 ng/mL)	Quartile 2 (~12.33 ng/mL)	Quartile 3 (~17.45 ng/mL)	Quartile 4 (>17.45 ng/mL)	*p* for Trend
General obesity (BMI)					
Model 1 ^a^	1 (ref)	1.14 (0.56, 2.35)	1.50 (0.76, 2.98)	1.51 (0.76, 3.00)	0.171
Model 2 ^b^	1 (ref)	1.14 (0.55, 2.36)	1.47 (0.73, 2.97)	1.38 (0.68, 2.81)	0.292
Central obesity (WC)					
Model 1 ^a^	1 (ref)	0.98 (0.66, 1.46)	1.28 (0.86, 1.88)	0.97 (0.65, 1.45)	0.767
Model 2 ^b^	1 (ref)	1.00 (0.66, 1.53)	1.33 (0.88, 2.02)	0.85 (0.56, 1.31)	0.772
Obesity (VFA)					
Model 1 ^a^	1 (ref)	0.82 (0.56, 1.19)	0.98 (0.67, 1.43)	0.69 (0.47, 1.00)	0.121
Model 2 ^b^	1 (ref)	0.81 (0.55, 1.20)	0.99 (0.67, 1.47)	0.64 (0.43, 0.95)	0.071
Obesity (BF%)					
Model 1 ^a^	1 (ref)	1.02 (0.70, 1.48)	0.97 (0.67, 1.40)	0.73 (0.50, 1.06)	0.096
Model 2 ^b^	1 (ref)	1.05 (0.71, 1.55)	0.98 (0.66, 1.46)	0.64 (0.43, 0.96)	0.034

Values are odds ratio (95% confidence interval). Abbreviations: BMI, body mass index; WC, waist circumference; VFA, visceral fat area; BF%, body fat percentage. ^a^ Model 1: unadjusted. ^b^ Model 2: adjusted for age, sex, marital status, education, smoking status, drinking status, employment status, physical activity, family income, daily energy intake (tertiles), nutritional supplement, and chronic diseases.

**Table 7:** Association between vitamin B_9_ and obesity according to different obesity definitions.

	VB_9_				
	Quartile 1 (<2.59 ng/mL)	Quartile 2 (~4.27 ng/mL)	Quartile 3 (~6.59 ng/mL)	Quartile 4 (>6.59 ng/mL)	*p* for Trend
General obesity (BMI)					
Model 1 ^a^	1 (ref)	1.12 (0.57, 2.17)	0.94 (0.47, 1.87)	1.18 (0.61, 2.28)	0.758
Model 2 ^b^	1 (ref)	1.11 (0.56, 2.18)	0.93 (0.46, 1.89)	1.16 (0.59, 2.27)	0.794
Central obesity (WC)					
Model 1 ^a^	1 (ref)	0.88 (0.60, 1.30)	0.86 (0.58, 1.28)	0.92 (0.62, 1.36)	0.656
Model 2 ^b^	1 (ref)	0.81 (0.53, 1.22)	0.84 (0.55, 1.27)	0.84 (0.56, 1.27)	0.464
Obesity (VFA)					
Model 1 ^a^	1 (ref)	0.74 (0.51, 1.08)	0.73 (0.50, 1.06)	0.66 (0.46, 0.97)	0.039
Model 2 ^b^	1 (ref)	0.67 (0.45, 0.99)	0.71 (0.48, 1.05)	0.61 (0.41, 0.91)	0.027
Obesity (BF%)					
Model 1 ^a^	1 (ref)	0.78 (0.54, 1.14)	0.77 (0.53, 1.12)	0.72 (0.49, 1.04)	0.090
Model 2 ^b^	1 (ref)	0.76 (0.51, 1.12)	0.76 (0.51, 1.13)	0.67 (0.45, 0.99)	0.057

Values are odds ratio (95% confidence interval). Abbreviations: BMI, body mass index; WC, waist circumference; VFA, visceral fat area; BF%, body fat percentage. ^a^ Model 1: unadjusted. ^b^ Model 2: adjusted for age, sex, marital status, education, smoking status, drinking status, employment status, physical activity, family income, daily energy intake (tertiles), nutritional supplement, and chronic diseases.

## Data Availability

The datasets used to support the findings of this study are available from the corresponding author upon reasonable request.

## Supplementary Materials

The following supporting information can be downloaded at: https://www.mdpi.com/article/10.3390/nu15030483/s1, Table S1: Association between vitamin B and VFA-defined obesity in older adults (≥60 years), Table S2: Association between vitamin B and WC-defined obesity in older adults (≥60 years), Table S3: Association between vitamin B and BF%-defined obesity in older adults (≥60 years), Table S4: Association between vitamin B and VFA-defined obesity in females, Table S5: Association between vitamin B and BF%-defined obesity in females, Table S6: Association between vitamin B and WC-defined obesity in females.
