# Supplementary material for: Association between Vitamin B and Obesity in Middle-Aged and Older Chinese Adults
Source: Nutrients. 2023 Jan 17;15(3):483. doi: 10.3390/nu15030483 (PMC9921635; doi:10.3390/nu15030483)
Supplement: Supplementary file 1 [file nutrients-15-00483-s001.zip › nutrients-2101173-supplementary.pdf]

Supplementary Online Content

**Supplement Table S1 Association between vitamin B and VFA-defined obesity in older adults (≥60 years)**

**Supplement Table S2 Association between vitamin B and WC-defined obesity in older adults (≥60 years)**

**Supplement Table S3 Association between vitamin B and BF%-defined obesity in older adults (≥60 years)**

**Supplement Table S4 Association between vitamin B and VFA-defined obesity in female**

**Supplement Table S5 Association between vitamin B and BF%-defined obesity in female**

**Supplement Table S6 Association between vitamin B and WC-defined obesity in female**

**Supplement Table S1 Association between vitamin B and VFA-defined obesity in older adults (≥60 years)**

| | OR (95% CI) [a] |
|---|---|
| $VB_1$ | |
| Quartile 1 (<2.60 ng/mL) | 0 (ref) |
| Quartile 2 (~3.16 ng/mL) | **0.54 (0.31, 0.94)** |
| Quartile 3 (~4.38 ng/mL) | **0.47 (0.27, 0.81)** |
| Quartile 4 (>4.38 ng/mL) | **0.47 (0.27, 0.84)** |
| $VB_2$ | |
| Quartile 1 (<2.98 ng/mL) | 0 (ref) |
| Quartile 2 (~4.58 ng/mL) | 0.71 (0.41, 1.23) |
| Quartile 3 (~7.18 ng/mL) | 0.98 (0.56, 1.70) |
| Quartile 4 (>7.18 ng/mL) | 0.58 (0.33, 1.00) |
| $VB_6$ | |
| Quartile 1 (<8.99 ng/mL) | 0 (ref) |
| Quartile 2 (~12.33 ng/mL) | 0.80 (0.46, 1.39) |
| Quartile 3 (~17.45 ng/mL) | 0.64 (0.36, 1.13) |
| Quartile 4 (>17.45 ng/mL) | **0.56 (0.32, 0.98)** |
| $VB_9$ | |
| Quartile 1 (<2.59 ng/mL) | 0 (ref) |
| Quartile 2 (~4.27 ng/mL) | 0.63 (0.36, 1.10) |
| Quartile 3 (~6.59 ng/mL) | 0.65 (0.37, 1.14) |
| Quartile 4 (>6.59 ng/mL) | **0.52 (0.30, 0.91)** |

[a] adjusted for sex, marital status, education, smoking status, drinking status, employment status, physical activity, family income, daily energy intake (tertiles), nutritional supplement and chronic diseases.

**Supplement Table S2 Association between vitamin B and WC-defined obesity in older adults (≥60 years)**

| | OR (95% CI) [a] |
|---|---|
| $VB_1$ | |
| Quartile 1 (<2.60 ng/mL) | 0 (ref) |
| Quartile 2 (~3.16 ng/mL) | 0.96 (0.57, 1.63) |
| Quartile 3 (~4.38 ng/mL) | 0.74 (0.44, 1.27) |
| Quartile 4 (>4.38 ng/mL) | **0.48 (0.27, 0.85)** |
| $VB_2$ | |
| Quartile 1 (<2.98 ng/mL) | 0 (ref) |
| Quartile 2 (~4.58 ng/mL) | 0.85 (0.50, 1.46) |
| Quartile 3 (~7.18 ng/mL) | 0.73 (0.42, 1.27) |
| Quartile 4 (>7.18 ng/mL) | **0.41 (0.23, 0.72)** |
| $VB_6$ | |
| Quartile 1 (<8.99 ng/mL) | 0 (ref) |
| Quartile 2 (~12.33 ng/mL) | 1.03 (0.59, 1.77) |
| Quartile 3 (~17.45 ng/mL) | 0.94 (0.53, 164) |
| Quartile 4 (>17.45 ng/mL) | 0.66 (0.38, 1.15) |
| $VB_9$ | |
| Quartile 1 (<2.59 ng/mL) | 0 (ref) |
| Quartile 2 (~4.27 ng/mL) | 0.99 (0.58, 1.71) |
| Quartile 3 (~6.59 ng/mL) | 0.82 (0.47, 1.42) |
| Quartile 4 (>6.59 ng/mL) | 0.65 (0.38, 1.14) |

[a] adjusted for sex, marital status, education, smoking status, drinking status, employment status, physical activity, family income, daily energy intake (tertiles), nutritional supplement and chronic diseases.

**Supplement Table S3 Association between vitamin B and BF%-defined obesity in older adults (≥60 years)**

| | OR (95% CI) [a] |
|---|---|
| $VB_1$ | |
| Quartile 1 (<2.60 ng/mL) | 0 (ref) |
| Quartile 2 (~3.16 ng/mL) | **0.52 (0.31, 0.91)** |
| Quartile 3 (~4.38 ng/mL) | **0.46 (0.27, 0.79)** |
| Quartile 4 (>4.38 ng/mL) | **0.41 (0.23, 0.72)** |
| $VB_2$ | |
| Quartile 1 (<2.98 ng/mL) | 0 (ref) |
| Quartile 2 (~4.58 ng/mL) | 0.61 (0.36, 1.05) |
| Quartile 3 (~7.18 ng/mL) | 1.02 (0.59, 1.76) |
| Quartile 4 (>7.18 ng/mL) | **0.56 (0.32, 0.96)** |
| $VB_6$ | |
| Quartile 1 (<8.99 ng/mL) | 0 (ref) |
| Quartile 2 (~12.33 ng/mL) | 1.02 (0.59, 1.76) |
| Quartile 3 (~17.45 ng/mL) | 0.66 (0.38, 1.16) |
| Quartile 4 (>17.45 ng/mL) | **0.57 (0.33, 0.99)** |
| $VB_9$ | |
| Quartile 1 (<2.59 ng/mL) | 0 (ref) |
| Quartile 2 (~4.27 ng/mL) | 0.68 (0.40, 1.17) |
| Quartile 3 (~6.59 ng/mL) | 0.61 (0.35, 1.06) |
| Quartile 4 (>6.59 ng/mL) | 0.59 (0.34, 1.02) |

[a] adjusted for sex, marital status, education, smoking status, drinking status, employment status, physical activity, family income, daily energy intake (tertiles), nutritional supplement and chronic diseases.

**Supplement Table S4 Association between vitamin B and VFA-defined obesity in female**

| | OR (95% CI) [a] |
|---|---|
| $VB_1$ | |
| Quartile 1 (<2.60 ng/mL) | 0 (ref) |
| Quartile 2 (~3.16 ng/mL) | 0.68 (0.45, 1.01) |
| Quartile 3 (~4.38 ng/mL) | **0.55 (0.37, 0.83)** |
| Quartile 4 (>4.38 ng/mL) | **0.52 (0.34, 0.79)** |
| $VB_2$ | |
| Quartile 1 (<2.98 ng/mL) | 0 (ref) |
| Quartile 2 (~4.58 ng/mL) | 0.88 (0.59, 1.32) |
| Quartile 3 (~7.18 ng/mL) | 1.03 (0.68, 1.54) |
| Quartile 4 (>7.18 ng/mL) | 0.73 (0.48, 1.10) |
| $VB_6$ | |
| Quartile 1 (<8.99 ng/mL) | 0 (ref) |
| Quartile 2 (~12.33 ng/mL) | 0.85 (0.57, 1.27) |
| Quartile 3 (~17.45 ng/mL) | 0.97 (0.65, 1.45) |
| Quartile 4 (>17.45 ng/mL) | **0.62 (0.41, 0.94)** |
| $VB_9$ | |
| Quartile 1 (<2.59 ng/mL) | 0 (ref) |
| Quartile 2 (~4.27 ng/mL) | 0.66 (0.44, 0.98) |
| Quartile 3 (~6.59 ng/mL) | 0.68 (0.46, 1.03) |
| Quartile 4 (>6.59 ng/mL) | **0.61 (0.40, 0.91)** |

[a] adjusted for sex, marital status, education, smoking status, drinking status, employment status, physical activity, family income, daily energy intake (tertiles), nutritional supplement and chronic diseases.

**Supplement Table S5 Association between vitamin B and BF%-defined obesity in female**

| | OR (95% CI) [a] |
|---|---|
| $VB_1$ | |
| Quartile 1 (<2.60 ng/mL) | 0 (ref) |
| Quartile 2 (~3.16 ng/mL) | 0.67 (0.45, 1.01) |
| Quartile 3 (~4.38 ng/mL) | **0.55 (0.37, 0.83)** |
| Quartile 4 (>4.38 ng/mL) | **0.49 (0.32, 0.75)** |
| $VB_2$ | |
| Quartile 1 (<2.98 ng/mL) | 0 (ref) |
| Quartile 2 (~4.58 ng/mL) | 0.67 (0.45, 1.00) |
| Quartile 3 (~7.18 ng/mL) | 0.93 (0.62, 1.40) |
| Quartile 4 (>7.18 ng/mL) | **0.64 (0.42, 0.97)** |
| $VB_6$ | |
| Quartile 1 (<8.99 ng/mL) | 0 (ref) |
| Quartile 2 (~12.33 ng/mL) | 1.01 (0.70, 1.51) |
| Quartile 3 (~17.45 ng/mL) | 0.95 (0.67, 1.43) |
| Quartile 4 (>17.45 ng/mL) | 0.70 (0.46, 1.06) |
| $VB_9$ | |
| Quartile 1 (<2.59 ng/mL) | 0 (ref) |
| Quartile 2 (~4.27 ng/mL) | 0.81 (0.54, 1.20) |
| Quartile 3 (~6.59 ng/mL) | 0.82 (0.55, 1.23) |
| Quartile 4 (>6.59 ng/mL) | 0.69 (0.46, 1.03) |

[a] adjusted for sex, marital status, education, smoking status, drinking status, employment status, physical activity, family income, daily energy intake (tertiles), nutritional supplement and chronic diseases.

**Supplement Table S6 Association between vitamin B and WC-defined obesity in female**

| | OR (95% CI) [a] |
|---|---|
| $VB_1$ | |
| Quartile 1 (<2.60 ng/mL) | 0 (ref) |
| Quartile 2 (~3.16 ng/mL) | 0.91 (0.60, 1.39) |
| Quartile 3 (~4.38 ng/mL) | 0.74 (0.48, 1.13) |
| Quartile 4 (>4.38 ng/mL) | **0.51 (0.32, 0.80)** |
| $VB_2$ | |
| Quartile 1 (<2.98 ng/mL) | 0 (ref) |
| Quartile 2 (~4.58 ng/mL) | 0.89 (0.58, 1.37) |
| Quartile 3 (~7.18 ng/mL) | 0.79 (0.51, 1.2) |
| Quartile 4 (>7.18 ng/mL) | 0.65 (0.42, 1.01) |
| $VB_6$ | |
| Quartile 1 (<8.99 ng/mL) | 0 (ref) |
| Quartile 2 (~12.33 ng/mL) | 0.95 (0.62, 1.47) |
| Quartile 3 (~17.45 ng/mL) | 0.97 (0.81, 1.92) |
| Quartile 4 (>17.45 ng/mL) | 0.83 (0.53, 1.30) |
| $VB_9$ | |
| Quartile 1 (<2.59 ng/mL) | 0 (ref) |
| Quartile 2 (~4.27 ng/mL) | 0.81 (0.53, 1.24) |
| Quartile 3 (~6.59 ng/mL) | 0.84 (0.54, 1.29) |
| Quartile 4 (>6.59 ng/mL) | 0.83 (0.54, 1.27) |

[a] adjusted for sex, marital status, education, smoking status, drinking status, employment status, physical activity, family income, daily energy intake (tertiles), nutritional supplement and chronic diseases.
